# Electrophysiological and behavioural responses to consonant and dissonant piano chords as standardised affective stimuli

**DOI:** 10.3389/fnhum.2025.1689067

**Published:** 2025-10-29

**Authors:** Alexander Kirsanov, Maria Koriakina, Denis Panasenko, Uliana Nikishkina, Evgeny Blagovechtchenski

**Affiliations:** 1 Laboratory of Behavioural Neurodynamics, Saint-Petersburg State University, Saint-Petersburg, Russia; 2 Affective Psychophysiology Laboratory, Institute of Health Psychology, National Research University Higher School of Economics (HSE), Saint-Petersburg, Russia

**Keywords:** chords, affective stimuli, ERP, source analysis, DFA, LRTC

## Abstract

Although the difference between consonance and dissonance has raised interest for decades in various fields of science, isolated chords are still underutilised as standardised affective stimuli in neuroscience. In the present study, we assessed whether consonant, dissonant, and neutral sounds evoked different subjective and neurophysiological responses associated with the emotional experience. For the first time, we conducted a comprehensive study of piano timbre chord perception, combining behavioural assessments, event-related potentials (ERPs), frequency-domain electroencephalography (EEG) analysis of amplitude, neurodynamic (longrange temporal correlations (LRTC)) factors, and source analysis. In an experiment, 30 participants rated the valence of sounds (consonance, dissonance, neutral) whilst simultaneously undergoing EEG recordings. At the behavioural level, a stable valence gradient was revealed: consonance was perceived as pleasant, dissonance as unpleasant, and the neutral group of stimuli as neutral. Analysis of ERPs revealed differences in response across three time windows (90–110 ms, 190–210 ms, 290–310 ms) and activation of frontotemporal and temporoparietal areas during the processing of dissonant chords. At the level of frequency and neurodynamic indices (gamma and beta bands amplitudes and LRTC), we demonstrated that classification accuracy depends on the interaction between chord type and the EEG’s amplitude- and scale-invariant characteristics for dissonant and neutral stimuli. These results provide evidence that isolated chords evoke differentiated emotional and cognitive responses, highlighting their potential utility as affective stimuli in future studies.

## 1 Introduction

The dichotomy between consonance and dissonance, originating in Pythagorean theory and subsequently reinterpreted through the Platonic prism of cosmic harmony (Lippman, 1994; Plato, 2019), remains a relevant theoretical origin underlying works ranging from music theory to cognitive neuroscience. Within this trajectory, musical intervals defined by simple frequency ratios (e.g., octaves, fifths) have been considered as pleasant due to their stability. Dissonant intervals, on the other hand, were characterised by more complex frequency relationships and were considered acoustically unstable, thus affecting the listener unpleasantly (Krumhansl and Jusczyk, 1990; Bidelman and Krishnan, 2009).

This differential perception of pleasant and unpleasant intervals is a specific manifestation of a broader neurophysiological mechanism for evaluating auditory stimuli. Indeed, emotional processing of distant stimuli, such as auditory or visual stimuli, is fundamental to the human experience and is associated with behavioural and physiological level reactions (Bradley and Lang, 2000; Keil et al., 2002). Negative auditory stimuli, such as screeching or screaming, typically evoke autonomic body responses attributed to the activation of the sympathetic nervous system (Kumar et al., 2012; Schweiger Gallo et al., 2017). Concrete sounds, however, are typically associated with specific, dangerous objects or circumstances related to traumatic episodes in a person’s life. Music, being more abstract than environmental sounds and rooted in culture, offers a different type of emotional impact that engages distributed networks, including limbic and default mode systems (Koelsch, 2010; Brattico et al., 2011; Schirmer, 2018).

Notably, affective research paradigms have long been dominated by visual stimuli, particularly static images, such as emotional faces or scenes (Lang and Bradley, 2010; Carretié, 2014; Dawel et al., 2022), and dynamic videos (Samson et al., 2016; Maffei and Angrilli, 2019). Whilst static images reliably evoke isolated unpleasant reactions (e.g., fear or disgust), videos induce higher arousal and more varied emotional responses (Horvat et al., 2015). However, in terms of temporal accuracy, ecological validity, and cultural specificity, both classes of visual stimuli have meaningful limitations (Boğa et al., 2023).

In contrast, auditory stimuli provide enhanced opportunities for cross-modal integration in experimental settings. Thus, amongst emotional primitives, consonance and dissonance are typically associated with pleasantness and unpleasantness, respectively (Fritz et al., 2009; Virtala and Tervaniemi, 2017; Harding et al., 2025). Auditory stimuli also elicit more stable and extensive autonomic responses when combined with visual stimuli (Song et al., 2021). In addition, validated auditory affective paradigms such as the International Affective Digitised Sounds (IADS) database (Bradley and Lang, 2000; Yang et al., 2018) and standardised music-emotion protocols (Juslin and Västfjäll, 2008; Koelsch, 2014) provide robust frameworks for studying affective auditory processing. Although less frequent than visual paradigms (Gerdes et al., 2014), these approaches highlight the critical role of auditory stimuli in emotion research.

Importantly, affective responses to consonance and dissonance agree with the findings of prosody research, in which the emotional characteristics of a speech stimulus are associated with variations in pitch and timbre (Schirmer and Kotz, 2006; Paulmann and Kotz, 2008; Liu et al., 2018). Both processes are based on mechanisms that rapidly evaluate acoustic characteristics, involving overlapping neural networks in the superior temporal and frontal cortices of the brain (Proverbio et al., 2020). In addition to these parallels, theoretical frameworks such as predictive coding characterise both music and speech as hierarchical systems where deviations from expected patterns—whether harmonic or prosodic—trigger prediction error signals (Friston, 2005; Koelsch et al., 2019). The shared syntactic integration resource hypothesis (Patel, 2003) suggests that there is a partially common pool of resources primarily linked to Broca’s area. Neuroimaging studies indicate that emotional prosody and dissonant harmonies activate overlapping frontal-temporal networks (Schirmer and Kotz, 2006; Koelsch, 2014; Proverbio et al., 2020). Furthermore, both limbic and auditory association areas are engaged by emotional vocalisations and harmonic expectancy (Frühholz et al., 2014). This similarity between emotional evaluation mechanisms for chords and prosodic speech signals highlights their potential as standardised stimuli for shared mechanisms of emotion across music and language.

Despite these advantages, the implementation of isolated musical chords within the domain of affective neuroscience remains limited. Chords represent a compact but powerful type of stimuli: parametrically driven, affectively salient, and ecologically relevant (in cases where a stimulus is required that is not associated with a specific object or situation). However, it remains debated whether these emotional labels correspond to genuine affective states or are the result of cognitive categorisation based on acoustic characteristics, such as frequency ratio or roughness (Gabrielsson, 2001; Eerola and Vuoskoski, 2011), and cultural experience (Parncutt and Hair, 2018). Recent meta-analyses of consonant and dissonant sounds suggest that simultaneous consonance is a complex phenomenon that largely stems from three key factors: interference, periodicity/harmonicity, and cultural familiarity. Additionally, the perceptual salience of dissonance has been also related to roughness as a marker of potential danger (Harrison and Pearce, 2020; see, also, Di Stefano et al., 2022).

Empirical studies have shown that harmonic structure modulates the auditory components of the event-related potentials (ERP): consonant stimuli often evoke an increase in N1 amplitude with latency around 100 ms, whereas dissonant stimuli are associated with an enhanced P2 response with latency around 200 ms, suggesting higher auditory coding demands (Regnault et al., 2001; Bidelman and Grall, 2014). Comparative studies have shown that both musicians and non-musicians exhibit early deviance-related potentials to unexpected chords such as MMN or its music-syntactic analogue ERAN (Koelsch et al., 2000; Vuust et al., 2012; Quiroga-Martinez et al., 2022). Taken together, N1, P2, and MMN represent well-established early ERP markers of auditory-affective processing, indexing rapid perceptual encoding, categorisation, and deviance detection that provide the neural basis for subsequent evaluative components. Notably, several studies have shown that these early components can display a “negativity bias,” with unpleasant or threatening stimuli eliciting stronger responses than arousal-matched positive ones (Carretié et al., 2004; Delplanque et al., 2006; Olofsson et al., 2008). Electrocorticographic (ECoG) recordings emphasise the sensitivity of the right superior temporal region to dissonance in the high gamma band between 75 and 200 ms post-stimulus onset (Foo et al., 2016).

The electrophysiological correlates of affective evaluation in response to musical stimuli are less well characterised than those underlying perceptual or syntactic processing. Most ERP studies have focused on amplitude rather than latency; arousal effects are typically observed at longer latencies, whereas findings on valence effects remain less consistent (Olofsson et al., 2008). Two main ERP components modulated by stimulus arousal are the early posterior negativity (EPN), with a peak on 200–300 ms after stimulus onset, which probably indicates the selection of emotionally arousing stimuli for further processing; this component has also been discussed in relation to predictive coding frameworks (Friston, 2005). The second one is the late positive potential (LPP), a slow-wave component associated with consolidation of emotional memory (Brown et al., 2012). Previously, LPP has been reported to be larger for negatively valenced stimuli (Ito et al., 1998).

Recent EEG and MEG studies on music perception demonstrate that chord sequences elicit specific ERP components related to both cognitive and affective evaluations. Thus, chords evaluated as ‘incorrect’ often elicit an early right anterior negativity (ERAN) with latency 150–200 ms after stimulus onset or later (≈250–280 ms) in some unpredictable-sequence paradigms, corresponding to pre-attentive neural responses to harmonic violations in Western tonal music, rather than subjective listener judgements (Ellison et al., 2015; Ishida and Nittono, 2022). Additionally, negative evaluations (‘dislike’ or ‘incorrect’) of musical chords are associated with centro-parietal P3b/P600 positivity in ERP signal around 400–600 ms, indicative of conscious cognitive evaluation. However, this remains distinct in the context of music perception. Finally, an LPP around 1,200 ms was observed, reflecting higher-order cognitive processes related to the conscious decision-making regarding musical correctness (in music harmony terms) or preference, rather than merely emotional arousal (Brattico et al., 2010; Zhang et al., 2022).

Additionally, in conjunction with ERP, another essential characteristic of the EEG that determines a reaction to external signals is the average brain activity within a particular frequency range of spectral activity oscillations, as well as its dynamics (Li et al., 2018). Since such activity of a specific person is relatively stable compared to the ERP, there is even a tendency to create a personal EEG profile (Sysoeva et al., 2023). Such a personal profile may be associated, amongst other things, with the individual’s characteristics and their reaction to specific emotional stimuli. There is evidence that different frequency bands of the brain are associated with particular brain functions. Although there is considerable discussion in the literature about this issue, is it possible to link a specific oscillation frequency to the functional activity of the brain? For example, previous studies have linked theta oscillations to affective processing and motivational salience (Miltner et al., 1999), alpha rhythms to inhibition and attentional control in emotion regulation, beta to the regulation of emotional responses (Balconi and Lucchiari, 2008), and gamma activity to emotional arousal (Li and Lu, 2009). In more detail, oscillatory brain activity in various EEG frequency bands provides meaningful insight into the temporal and functional dynamics of emotional information processing. According to recent reviews, slow delta (0.5–4 Hz) and theta (4–7 Hz) rhythms are increased when a stimulus has high motivational significance and are associated with emotion regulation. Frontal theta oscillations increase during cognitive control in conditions of emotional conflict and successful re-evaluation of the situation (Knyazev, 2007; Ertl et al., 2013; Güntekin and Başar, 2014). Alpha activity (8–13 Hz) reflects the mechanisms of inhibition and attention distribution: a decrease in alpha power in the frontal lobes corresponds to high cortical activation during an emotional task, and frontal asymmetry of the alpha rhythm (FAA; ln R – ln L) correlates with approach and avoidance motivation, although the reliability of this index varies between studies (Davidson, 1998; Allen et al., 2004; van der Vinne et al., 2017). Beta rhythms (13–30 Hz), associated with arousal and motor readiness, show an increase in both anxiety states and increased emotional control (Miskovic and Schmidt, 2010; Schubring and Schupp, 2021). It is important to note that the functional significance of beta activity in emotional contexts remains a subject of debate. The literature reports seemingly contradictory findings, where beta power has been associated with processes ranging from anxiety and emotional regulation (Miskovic and Schmidt, 2010) to sensory integration and cognitive evaluation (Schubring and Schupp, 2021). This variability suggests that beta oscillations are not a univariate marker of a single process such as anxiety but may reflect a broader mechanism of cortical control or active inhibition during salient perceptual events. Gamma activity (>30 Hz) increases during highly arousing emotional stimuli and reflects integrative evaluative processing in occipito-temporal and limbic regions (Keil et al., 2002; Balconi and Lucchiari, 2008; Strube et al., 2021). Recent evidence suggests that enhancement of gamma potentials during the presentation of affective stimuli may be associated with improved subsequent memory, particularly for highly arousing negative scenes (Theódórsdóttir and Höller, 2023). However, this finding has thus far been reported primarily in the context of visual paradigms, and further research is needed to establish whether it generalises to auditory affective stimuli. Additionally, Güntekin and Başar (2014) provided a narrative overview of oscillatory responses to emotional visual stimuli, highlighting frequency-specific modulations but without applying a systematic methodology. Moreover, over the past few decades, new methods for assessing the neurodynamics of various rhythms have been developed, such as a method for analysing long-term correlations in the EEG signal (Sangiuliano Intra et al., 2018). This method has a physical basis, since it reflects the excitation/inhibition ratio, as well as the efficiency of information processing in the nervous system (Nikulin and Brismar, 2004, 2005). The relationship between long-range temporal correlations (LRTC) and emotions can be specific to certain EEG frequency bands (e.g., theta, alpha, beta), with different bands potentially reflecting various aspects of emotional processing (Bornas et al., 2015). In this study, we also assessed the emotional perception of different chords based on the frequency analysis of the subject’s EEG spectrum and the neurodynamics (LRTC) of the main rhythms.

This study aims to expand on existing work by employing a comprehensive methodology, with a particular focus on two aspects: first, the underutilization of auditory consonant stimuli, particularly chords, as affective stimuli; and second, the relatively modest integration of such stimuli into multimodal affective neuroscience research. We hypothesise that isolated musical chords will elicit reliable behavioural and electrophysiological markers of emotion—specifically, differentiated self-reported valence/arousal ratings and modulations of emotion-sensitive ERP components such as early components (N1, N2, P3). We also employed an analysis that combined behavioural categorisation with EEG recordings for source localisation (sLORETA) to investigate the early and late stages of affective auditory processing of consonant and dissonant chords. Based on previous data (Pascual-Marqui, 2002), we hypothesised that emotional chords would evoke enhanced P2 and P3a components compared to neutral sounds, reflecting their higher emotional salience and attention capture. We also assume that a person’s response to certain stimuli may be associated with different EEG frequency ranges, both in their amplitude and neurodynamic components (LRTC). By considering consonance and dissonance within the broader framework of affective neuroscience and grounding our predictions in psychophysiological and cultural theory, this study proposes musical chords as compact, emotionally relevant, and experimentally accessible stimuli for studying the neurocognitive architecture of affect.

## 2 Materials and methods

### 2.1 Participants

Thirty healthy non-musicians (mean age = 25.6 years, SD = 8, range = 18–45, 21 females) participated in the study. All had no history of hearing problems, speech disorders, neurological or psychiatric conditions, and no use of medications affecting brain function, as confirmed by a brief screening questionnaire. Participants’ musical experience was assessed in a short, structured oral interview. They were asked to report (i) whether they had ever received formal musical training, (ii) the instrument(s) played, (iii) the total number of years of instruction, (iv) whether they were currently practising or performing, and (v) the approximate frequency of musical activity in the past 5 years. Participants were not considered as musicians if they had less than 2 years of musical education and had not engaged in regular rehearsals (defined as more than 1 h per week) during the previous 5 years. These thresholds are broadly consistent with prior studies (e.g., Koelsch et al., 2000; Bidelman and Krishnan, 2009). All participants gave their written consent to participate in the experiment. The Ethics Committee of St. Petersburg State University approved the study.

### 2.2 Experimental task and procedure

In the present study, auditory stimuli were constructed using piano tones from the C₃–B₃ range (130.8–246.9 Hz, traditionally referred to as the ‘small octave’) and from the C₄–B₄ range (261.6–493.9 Hz, traditionally referred to as the ‘one-line octave’). All chords were constructed in accordance with the method described by Steinbeis and Koelsch (2011) and were used as separate individual chord sounds. The consonant chords consisted of seven major triads in root position, presented in the configuration I–V–I (C–E–G–C, D–F#–A–D, E–G#–B–E, F–A–C–F, G–B–D–G, A–C#–E–A, B–D#–F#–B) and seven six-four inversions of these chords in the V–I–V position (e.g., G–C–E–G for C major, A–D–F#–A for D major).

Dissonant chords were constructed by superimposing fixed interval patterns on the root notes of the natural diatonic scale (C, D, E, F, G, A, B). Two types of dissonant structures were used. The first consisted of the root, augmented fourth (+6 semitones), perfect fourth (+5 semitones), and minor second (+1 semitone), for example: C–F#–B–C, D–G#–C#–D, E–A#–D#–E. The second consisted of the root, minor second (+1 semitone), perfect fourth (+5 semitones), and augmented fourth (+6 semitones), for example, C–C#–F#–C, D–D#–G#–D, E–F–A–E. These interval structures were designed to maximise sensory dissonance through the proximity of sounds and non-harmonic intervals. All stimuli consisted of isolated chords defined by their intervallic content, with no harmonic progression intended or implied.

All sounds were generated using GarageBand software (Apple Inc., Cupertino, CA, United States). All chord stimuli had a duration of 800 ms and were normalised in amplitude using GarageBand software. They were synthesised with the same timbre (Grand Piano preset). The neutral auditory control consisted of single metronome clicks. In affective ERP paradigms, neutral stimuli are typically operationalised as low-complexity baseline conditions (Bradley and Lang, 2000; Olofsson et al., 2008) that minimise emotional salience. In line with this principle, we chose metronome clicks, which are acoustically simple, culturally familiar, and free of harmonic content. These clicks were synthesised using a sampler and a pre-recorded metronome sound. Each digitally metronome sound was modulated in pitch to correspond to a tone within the C₃–B₄ range (the small and one-line octaves). Auditory stimuli were presented via semi-open headphones (AKG K-240 mk2, Harman International Industries) at an individually adjusted, subjectively comfortable volume level.

**Figure 1:**
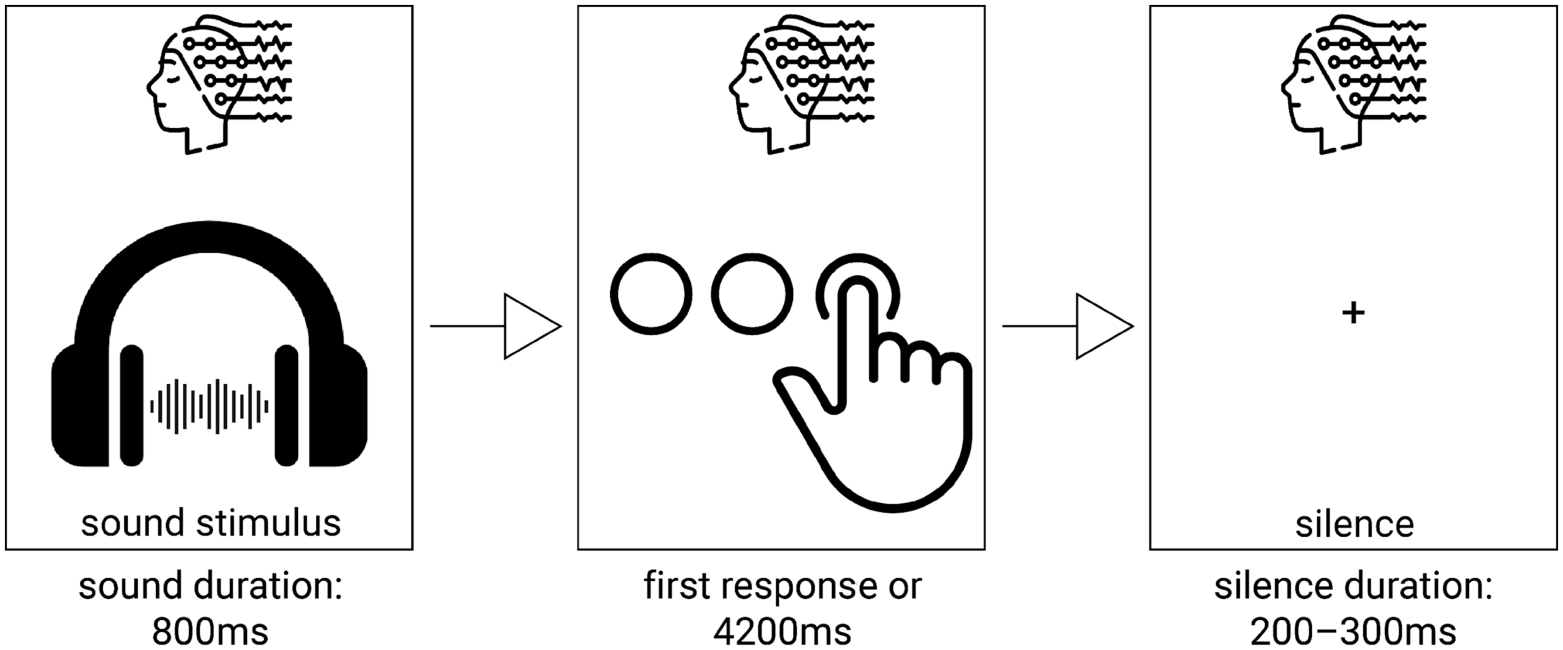
Experimental procedure of auditory stimuli presentation.

Throughout the experimental task (Figure 1), a fixation cross remained continuously displayed at the centre of the screen to maintain participants’ visual attention and minimise eye movements. Auditory stimuli were presented separately with 200–300 ms interval between stimuli via high-quality stereo headphones, ensuring balanced binaural delivery. Each auditory stimulus was presented five times across the session in a randomised order, whilst the visual fixation cross was presented on the screen, the total duration of the experimental session did not exceed 20 min. Following each stimulus presentation, participants were instructed to categorise their subjective impression of the sound as pleasant, unpleasant, or neutral. Responses were provided using the index finger of the right hand on a response pad (RB-740, Cedrus Inc.), with each key corresponding to one of the three response options (neutral, pleasant or unpleasant). Throughout the task, continuous recordings of brain activity with EEG, reaction times, and behavioural responses were collected for subsequent analysis. At the onset of each auditory stimulus, a time-locked event marker was sent to the EEG recording system, allowing for the precise synchronisation of neural and behavioural data.

### 2.3 Recording

EEG data were recorded using a BrainVision Recorder (BrainProducts GmbH, Gilching, Germany) with a 64-channel LiveAmp (BrainProducts GmbH, Gilching, Germany) system and an R-Netcap (BrainProducts GmbH, Gilching, Germany) system. The FCz electrode was used as the reference. Electrode placement followed the extended 10–20 system (M1 montage; EasyCap, Herrsching, Germany), with all impedances kept below 25 kΩ. Signals were sampled at 500 Hz without online filtering. During the experimental procedure, participants were sitting in a comfortable, adjustable chair inside a sound-attenuated, temperature-controlled, and shielded room.

### 2.4 EEG analysis

#### 2.4.1 EEG preprocessing

EEG data were preprocessed using a custom MATLAB-based pipeline (R2022b, MathWorks, Natick, MA, United States), in combination with the Berlin Brain-Computer Interface (BBCI) (https://github.com/bbci) toolbox. EEG recordings were first downsampled to 250 Hz to optimise preprocessing (e.g., ICA) and reduce computational load, without loss of information in the analysed frequency range (≤45 Hz). A zero-phase 2nd-order Butterworth band-stop filter (45–55 Hz) was applied as a notch filter, followed by a band-pass filter (1–45 Hz). Data were subsequently re-referenced to the common average reference.

Noisy channel detection was performed through spectral power analysis and visual inspection, with channels exhibiting excessive noise (absolute amplitude greater than 100 μV). Independent Component Analysis (ICA) was conducted using FastICA (Hyvarinen, 1999). Components associated with ocular artefacts were identified by their characteristic frontal topography and temporal correlation with blink events and subsequently removed from the data.

Preprocessed EEG data were segmented into epochs time-locked to stimulus onset (−100 to 800 ms) and baseline-corrected using the −100 to 0 ms pre-stimulus interval.

#### 2.4.2 Source analysis

Finally, given the observed differences in ERP amplitudes across chord types, we computed the neural generators underlying this ERP activity using a distributed source reconstruction approach. Specifically, an sLORETA solution (Pascual-Marqui, 2002) was calculated on a realistic head model based on the Montreal Neurological Institute (MNI) template, employing a boundary-element method to account for the conductivity properties of head tissues. Source reconstruction was constrained to cortical grey matter and applied to grand-averaged data to enhance signal-to-noise ratio. The analysis was performed using the sLORETA/eLORETA software package on the mean ERP of dissonant and neutral conditions within the peak time intervals identified in the signal-space ERP analysis. sLORETA has a fundamental limitation in its low spatial resolution, but with the number of electrodes 64 is enough to estimate source localisation.

#### 2.4.3 Amplitude analysis and long-range temporal correlations

To assess the amplitude and neurodynamics of the main EEG rhythms, the entire EEG recording was used throughout the experiment. The amplitude of the EEG signal in each frequency range was estimated using the following algorithm: the signal was filtered in certain frequency ranges (alpha: 8–12 Hz, beta: 20–25 Hz, gamma: 35–40 Hz) using a two-way Butterworth bandpass filter. For the resulting signal, an envelope curve was constructed based on the Gilbert transform. To assess the amplitude of each rhythm, the average value of the envelope curve was found. To evaluate the neurodynamic characteristics, detrended fluctuation analysis (DFA) was employed, which reflects the LRTC with the window length varying from 5 to 50 s distributed equidistantly on a logarithmic scale (the algorithm for this analysis is described in Nikulin and Brismar, 2004).

#### 2.4.4 Statistical analysis

We applied Fisher’s exact test (Fisher, 1992) to examine the association between stimulus type and response category. To compare reaction times (RT) across the six groups formed by stimulus type (consonant, dissonant, neutral) and response congruency (congruent vs. incongruent), we then used a Kruskal–Wallis rank-sum test (Kruskal and Wallis, 1952). In all pos-hoc analysis Bonferroni correction was used.

All EEG/ERP analyses were conducted using custom scripts developed in the MATLAB 12.0 environment (MathWorks, Natick, MA), in combination with the BBCI toolbox. To address the issue of multiple comparisons arising from tests conducted across numerous channels, statistical significance for both the Wilcoxon signed-rank tests (Wilcoxon, 1945) and Spearman correlations (Spearman, 1904) was assessed across subjects using cluster-based permutation methods (Maris and Oostenveld, 2007). The permutation statistics is a method for multiple comparison analysis in electrode space. Eight hundred permutations were conducted for each contrast, with a two-tailed significance threshold set at *p* < 0.05. Cluster-level statistics were corrected for multiple comparisons using the family-wise error rate (FWER), allowing for robust inference on the spatiotemporal patterns of EEG activity.

Generalised linear mixed-effects models (GLMMs) with a binomial logit linkage (Hensher and Greene, 2003) were used to assess how oscillatory neural activity modulates affective categorisation. GLMMs are a flexible statistical framework for analysing binary outcomes. They allow the inclusion of both continuous and categorical predictors, whilst considering random effects such as variability at the participant and stimulus levels. The models included fixed effects for stimulus type (consonant, neutral, dissonant), oscillation dynamics in relevant frequency bands (DFA and frequencies), and their interactions. To account for repeated measures and individual variability, the model specified random intercepts and slopes for stimulus type across participants, and random intercepts across items (i.e., unique chord stimuli).

This modelling approach allowed us to test whether the probability of categorising stimuli as *a priori* groups (consonant as pleasant, dissonant as unpleasant, neutral as neutral) changes as a function of stimulus type and neural dynamics during the task. We used likelihood ratio tests to compare full models with reduced (null) models and calculated odds ratios with 95% confidence intervals from fixed effects to simplify interpretation.

In our interpretation, we focused on interaction effects directly from the retrieved models. This decision was based on current best practises in statistical modelling of neurocognitive data, emphasising the interpretability and simplicity of theoretically based models (Barr et al., 2013; Schad et al., 2020).

We did not apply a global correction across all analyses (ERP, oscillatory, LRTC, and behavioural); the results should therefore be interpreted accordingly.

## 3 Results

### 3.1 Behavioural results

Behavioural results showed significant differences in response distributions across consonant, dissonant, and neutral auditory stimuli. Current analysis was based on predefined expected responses: “pleasant” for consonant chords, “unpleasant” for dissonant chords, and “neutral” for metronome sounds.

**Figure 2:**
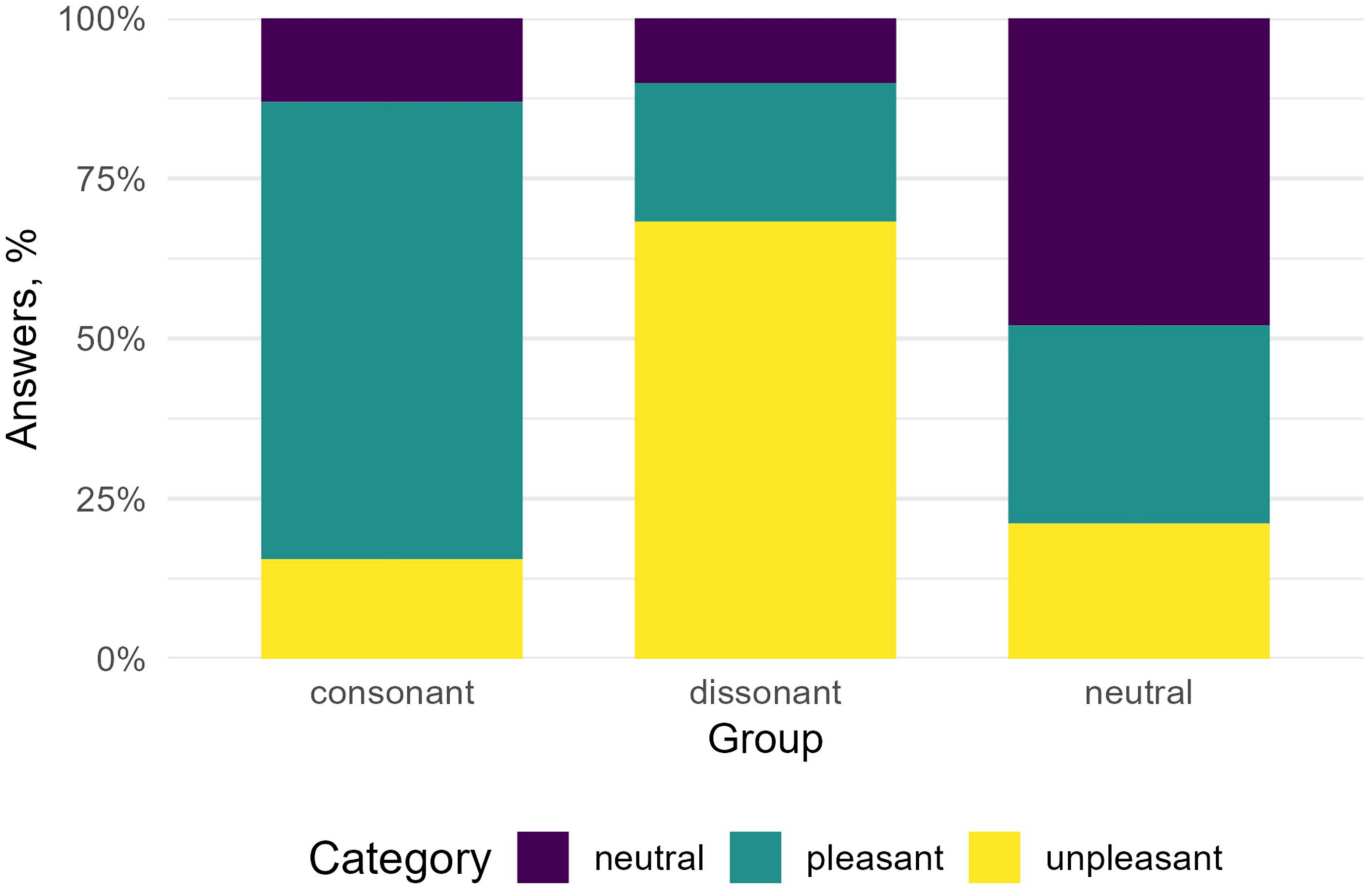
Proportion of “pleasant,” “neutral,” and “unpleasant” responses in categorisation task for dissonant, consonant, and neutral chords.

The analysis demonstrated a statistically significant association between stimulus type and response classification (*p* < 0.001), indicating that the distribution of correct and incorrect responses varied systematically across stimulus categories (Figure 2). These results suggest that participants statistically differentiate the emotional valence of consonant, dissonant, and neutral auditory stimuli with statistical significance.

A Kruskal–Wallis rank sum test revealed a significant difference in RT between groups, χ^2^(5, *N* = 4,702) = 261.99, *p* < 0.001. Post-hoc pairwise comparisons using Wilcoxon rank-sum tests with Bonferoni correction showed significant differences (adjusted *p* < 0.05) between most conditions, except for comparisons between dissonant and consonant in each congruent and incongruent condition, which were not statistically significant. Mean reaction times were as follows: dissonant congruent—522 ms, consonant congruent—464 ms, neutral congruent—747 ms, dissonant incongruent—1,123 ms, consonant incongruent—1,158 ms, neutral incongruent—688 ms.

### 3.2 EEG results

**Figure 3:**
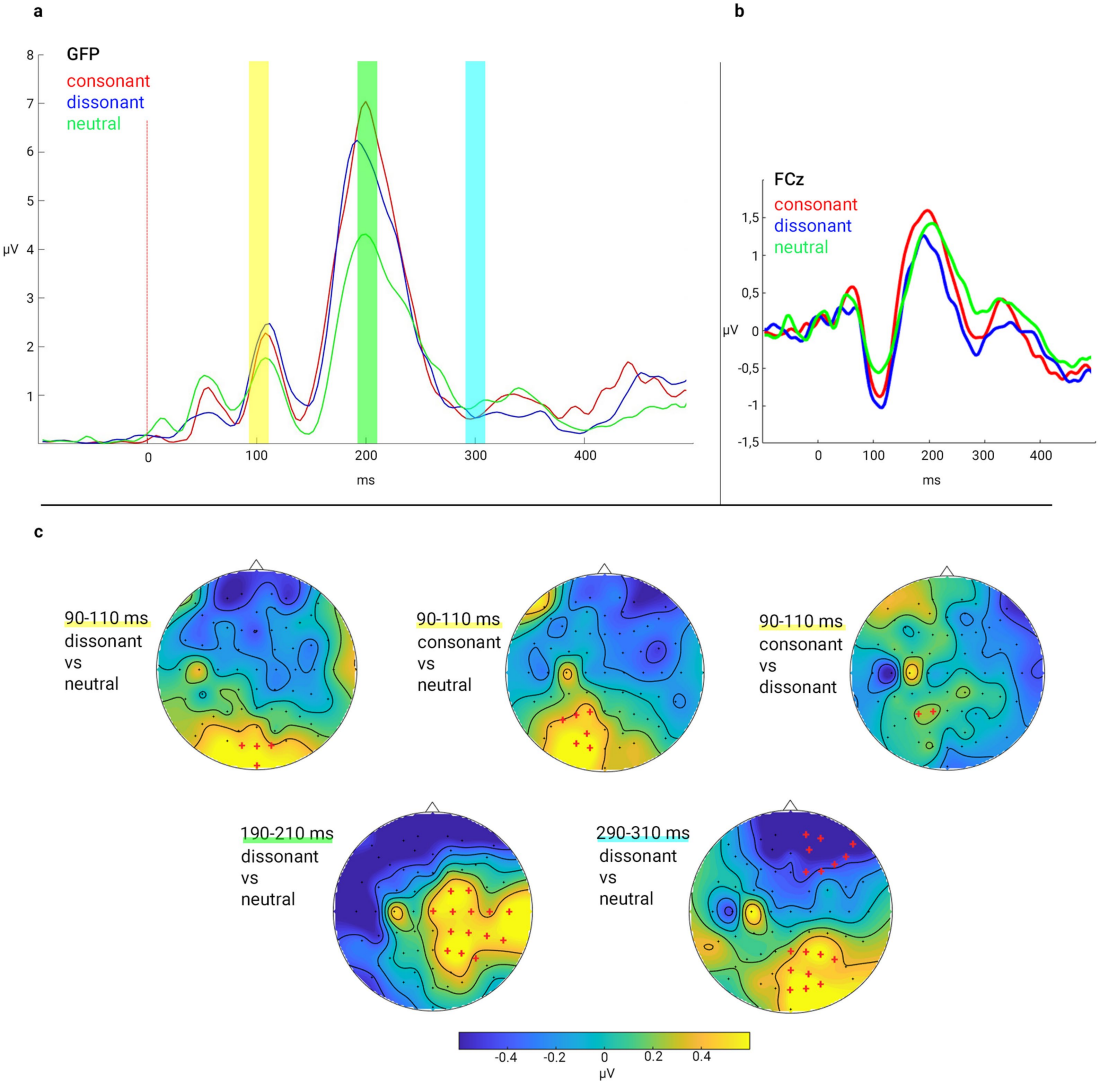
EEG results: **(a)** Global field power (GFP) of the ERP responses across all scalp electrodes for consonant, dissonant and neutral audio stimuli with three time-windows of interest 90–110, 190–210, and 290–310 ms after stimulus onset; **(b)** ERP responses in FCz electrode for consonant, dissonant and neutral audio stimuli; **(c)** Results of permutation analysis is shown clusters of electrodes with statistically significant differences between ERP responses in different stimulus types in considered time windows. Red crosses indicate channels belonging to a significant cluster (*p* < 0.05). The coloured scale (in μV) on the figure represents the gradient of the difference on the topograms.

A global field power transformation (GFP) was performed across all stimuli, conditions, and volunteers to objectively quantify the overall response pattern and identify time windows for further analysis. The resulting GFP curve showed three most prominent peaks corresponding to classical ERP components with maxima at ~100, 200, and 300 ms. Time windows of 90–110 ms, 190–210 ms, and 290–310 ms post-stimulus onset were identified as regions of interest based on the timing of overall neural activity (Figures 3A,B).

Permutation analysis (Figure 3C) was used to identify significant differences in brain activation between stimulus groups: consonant versus neutral chords, dissonant versus neutral chords, and consonant versus dissonant chords. Difference curves were calculated for each pair of conditions, followed by permutation analysis to determine statistically significant activation differences in the selected time windows.

In the earliest time window (90–110 ms), comparisons revealed distinct scalp activation patterns for different stimulus conditions. The contrast between consonant and neutral chords revealed significant differences, predominantly in the posterior-central and right anterior-central scalp regions. Comparing dissonant chords with neutral chords identified localised significant differences in posterior occipital scalp areas. Additionally, consonant versus dissonant chord comparisons demonstrated moderate but focal significant differences in the right temporal and left central scalp regions. In the time window 190–210 ms, dissonant chords compared to neutral chords elicited widespread significant activation differences, predominantly observed over bilateral posterotemporal and right frontocentral scalp regions. In the time window 290–310 ms, comparisons between dissonant and neutral chords revealed extensive and significant activation differences distributed broadly across bilateral frontal, temporal, and occipital scalp areas.

**Figure 4:**
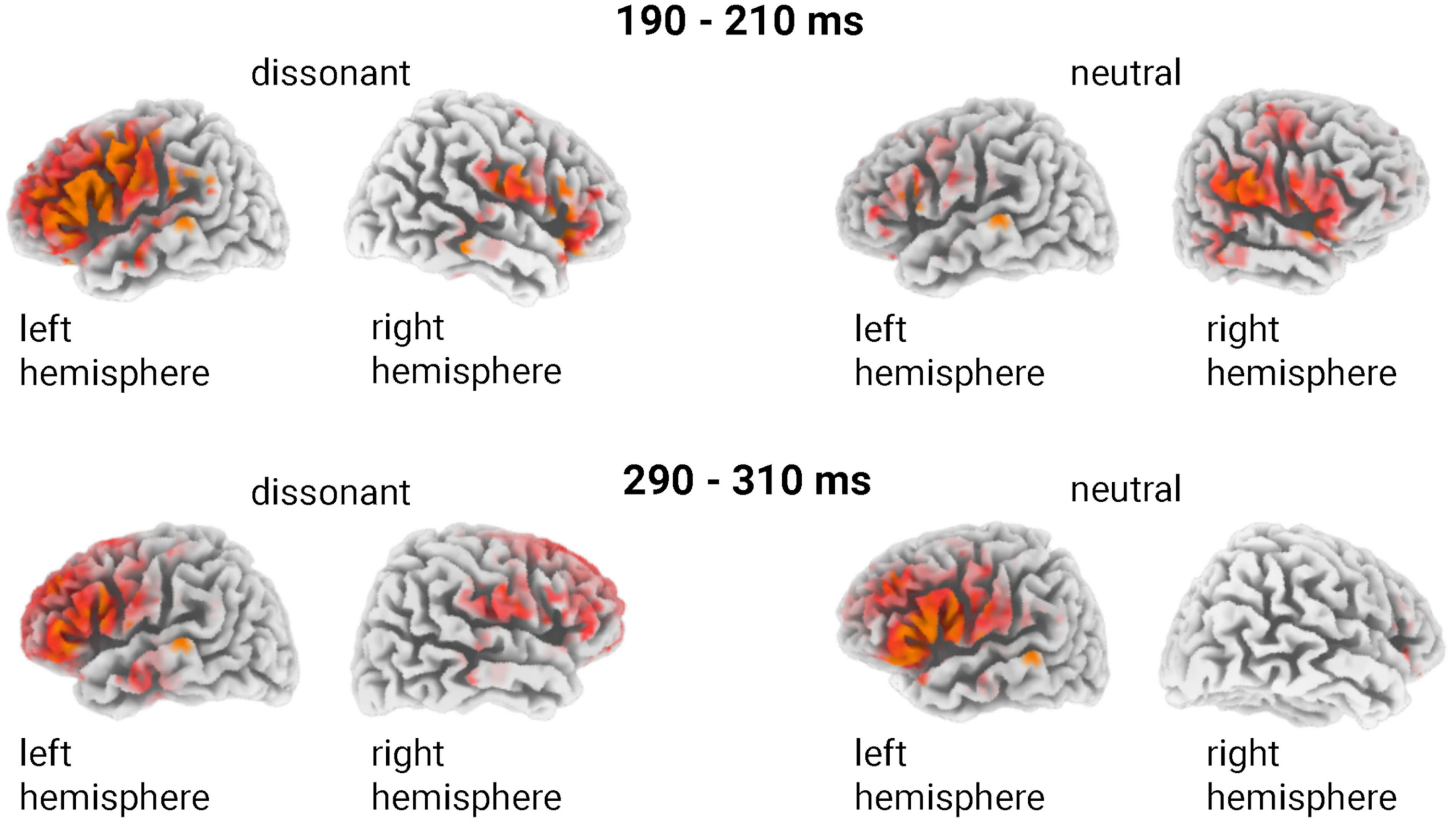
Cortical source activation contrasts between dissonant and neutral auditory stimuli across two analysed time windows (190–210 ms and 290–310 ms).

To further localise and interpret the neural generators of these ERPs, sLORETA analysis was conducted (Figure 4). Source localisations were assessed separately for dissonant and neutral stimulus types across the two latest time windows. As the analysis was exploratory and the spatial precision of sLORETA is limited, no quantitative peak values or coordinates are reported.

Brain activation observed at the first time window (90–110 ms) was excluded from the detailed analysis due to its relatively small magnitude differences and limited relevance to the emotional processing objectives of this study. At the earlier interval (190–210 ms), dissonant stimuli elicited enhanced cortical activation predominantly in the right frontotemporal and bilateral posterior temporoparietal areas compared to neutral stimuli, indicating increased cognitive and affective processing specifically associated with dissonance perception. During the later interval (290–310 ms), the response to dissonant stimuli further intensified, resulting in widespread bilateral frontal and temporal activation suggestive of heightened attentional engagement and emotional salience. Notably, the pattern of source lateralisation evolved from predominantly right-hemispheric during the earlier stage to symmetrical bilateral involvement in the later processing stage.

### 3.3 Mixed-effects logistic regression: oscillatory predictors of accuracy

To assess the relationship between trial-level categorisation accuracy and oscillatory brain activity, we fitted a series of mixed-effects logistic regression models. Each model included fixed effects for stimulus type (consonant, dissonant, neutral) and a continuous neural predictor (either group-specific amplitude or LRTs based on DFA), as well as their interaction. Random intercepts were set for stimuli, and random intercepts and uncorrelated slopes for stimulus type were included for participants. The significant and marginal effects found in the mixed-effects logistic models are presented in Table 1. Full details of the additional model analyses are provided in Supplementary materials S4–S7.

**Table 1:** Significant and marginal effects from mixed-effects logistic models predicting classification accuracy.

Band	Predictor	β	SE	*z*	*p*	OR [95% CI]
Beta (20–25 Hz)	Dissonant × amplitude	−2.08	0.94	−2.22	0.027	0.12 [0.02, 0.78]
Gamma (35–40 Hz)	Dissonant	2.13	0.84	2.52	0.012	8.41 [1.6, 44]
Gamma (35–40 Hz)	Dissonant × amplitude	−4.37	1.49	−2.93	0.003	0.01 [0.00068, 0.23]
Beta DFA	Neutral × DFA	−8.1	4.13	−1.96	0.050	<0.001 [9.1e-08, 1]
Gamma DFA	Neutral	4.88	02.03	2.4	0.016	131.63 [2.4, 7100]
Gamma DFA	Neutral × DFA	−9.74	03.07	−3.17	0.002	<0.001 [1.4e-07, 0.024]

The first column represents the frequency band used in the analysis; the predictor column includes data on significant interactions; SE denotes standard error; OR, odds ratio; CI, confidence interval.

#### 3.3.1 Amplitude-based models

In the alpha band (8–12 Hz), neither the main effect of amplitude nor the interaction with stimulus type reached statistical significance (all *p* < 0.05).

In the beta band (20–25 Hz), a significant interaction was found between beta amplitude and dissonant stimuli (β = −2.08, SE = 0.94, *z* = −2.22, *p* = 0.027, OR = 0.12 [0.02, 0.78]), indicating that increased beta amplitude was associated with reduced classification accuracy for dissonant chords.

In the gamma band (35–40 Hz), dissonant stimuli were more likely to be correctly classified when gamma amplitude was low (β = 2.13, SE = 0.84, *z* = 2.52, *p* = 0.012, OR = 8.41 [1.6, 44]), but this effect reversed at higher gamma amplitudes (β = −4.37, SE = 1.49, *z* = −2.93, *p* = 0.003, OR = 0.01 [0.00068, 0.23]).

#### 3.3.2 DFA-based models

In the alpha band DFA model, no main effects or interactions were statistically significant (all *p*s ≥ 0.12).

For beta DFA (20–25 Hz), classification accuracy was higher for neutral stimuli (*p* = 0.074), although this advantage diminished at higher beta DFA values (β = −8.10, SE = 4.13, *z* = −1.96, *p* = 0.050, OR < 0.001 [9.1e-08, 1]).

In the gamma DFA logistic regression model (35–40 Hz), neutral chords were more accurately classified at low DFA values (β = 4.88, SE = 2.03, *z* = 2.40, *p* = 0.016, OR = 131.63 [2.4, 7100]) [an extreme odds ratio followed by a very wide confidence interval reflects an imbalance in the number of correct and incorrect responses in this type of presented material and we interpret this outcome cautiously, focusing on the direction of the association (OR > 1)]; however, this effect was reversed with increasing gamma DFA (β = −9.74, SE = 3.07, *z* = −3.17, *p* = 0.002, OR < 0.001 [1.4e-07, 0.024]).

## 4 Discussion

The current study investigated whether isolated musical chords in piano timbre, differing in harmonic structure (consonant, dissonant, or neutral), or neutral metronome clicks evoke differentiated affective responses in non-musicians at both behavioural and neurophysiological levels, including ERP (with source analysis), frequency, and neurodynamics analysis.

Our results showed differences in behavioural valence ratings: consonant chords were rated as more pleasant. In contrast, dissonant chords were rated as more unpleasant, whereas neutral metronome clicks were more often rated as neutral; thus supporting a reliable distinction between consonant and dissonant chords and metronome clicks in subjective valence ratings. Consonant chords were rated as more pleasant than dissonant chords, which, in turn, were rated as more unpleasant. These results replicate earlier findings that consonance and dissonance are categorised as pleasant or unpleasant, respectively (Gabrielsson, 2001; Bidelman and Krishnan, 2009; McDermott et al., 2010). Furthermore, in our study, consonant and dissonant chords differed in their ratings from neutral auditory stimuli, indicating a clear differentiation of the selected auditory stimuli. It is noteworthy that the average ratings of neutral stimuli indicate that listeners did not rely on binary judgements but differentiated stimuli according to valence gradation. The precise pattern of subjective differentiation obtained even amongst non-musician listeners supports the idea that isolated harmonic structures alone are sufficient for evaluative affective judgements. However, it should be noted that previous studies have demonstrated that listeners can make valence judgements based on acoustic features such as roughness or interval size without necessarily experiencing an affective state (Gabrielsson, 2001; Juslin and Laukka, 2004).

To investigate the experience of emotions, an EEG analysis was conducted. At the electrophysiological level, different ERP components were modulated by chord type: all three sound types differed at the level of primary auditory processing, and at later stages of processing, dissonant chords evoked greater P2 and P3a amplitudes compared to neutral stimuli, indicating increased attentional and affective salience. Consider these results in more detail during the P1/N1 window (90–110 ms), where significant differences in brain substrate activation were observed between all three types of stimuli. Such an early difference may reflect low-level encoding of tonal characteristics. Previously, it was found that both the brainstem, as indicated by frequency-following responses (FFR), and early ERP components of the cerebral cortex, such as N1, are sensitive to harmonic structure (Bidelman and Krishnan, 2009; Bidelman, 2013; Bianco et al., 2016; Kurt Steinmetzger, 2024). These early components occur automatically and indicate the processing of low-level acoustic features before conscious cognitive evaluation can take place. Our results partially support Bianco et al. (2016) finding, which demonstrated that consonant intervals evoke earlier and stronger responses in the auditory cortex compared to dissonant intervals, consistent with the perceptual superiority of consonance in early stages of processing.

In the 190–210 ms time window, dissonant chords evoked significantly broader activation than neutral stimuli, especially in bilateral posterior temporal and right frontocentral areas. This activation pattern corresponds to the P2 component (Schirmer and Kotz, 2006; Paulmann and Kotz, 2008; Schirmer, 2018). Dissonant stimuli typically evoke greater cognitive effort and affective engagement, and we suggest that the observed higher cortical activity may reflect this process. ERP studies have shown that significant differences are observed in this time window for pitch in musicians and the perception of roughness in non-musicians (Kung et al., 2014). That is, already at an early stage of processing, the acoustic properties of stimuli significantly influence their categorisation by a brain substrate.

In the 290–310 ms time window, dissonant stimuli evoked neural responses in the frontal, temporal, and occipital regions. This activity is associated with the P3 family component, which is typically linked to the automatic reorientation of attention to emotionally significant or novel stimuli. Previous studies of musical and emotional prosody have shown that incongruent or emotionally charged auditory stimuli reliably enhance the amplitude of P3 (Koelsch, 2010; Proverbio et al., 2020). The fact that presented musical stimuli evoked a pattern similar to affective vocal expressions supports the view that music and speech share common pathways for affective processing (Koelsch, 2014). These results suggest that dissonant chords, due to their affective significance, inherently attract attention and evoke an orienting response.

The ERP responses described above—characterised by well-defined N1, P2, and P3 components—closely resemble findings reported in the literature on emotional stimuli across multiple sensory modalities: emotional prosody and affective non-verbal sounds (e.g., screams, laughter, crying) elicit early modulation in components such as N1 and P2, underscoring the automatic and pre-attentive analysis of emotional value (Schirmer and Kotz, 2006; Paulmann and Kotz, 2008). Likewise, previous studies on emotional visual stimuli, such as negative, arousing pictures from standardised image sets such as the International Affective Picture System (IAPS) or EmoMadrid (Carretié et al., 2019), have reported an increase of early posterior negativity (EPN) at 200–300 ms and amplified late positive potentials (LPP), indicative of sustained attentional and evaluative processing (Olofsson et al., 2008; Hajcak et al., 2010; Carretié, 2014). This finding supports the hypothesis that partially shared neural mechanisms are involved in processing emotional salience in both music and language (Ramaswamy and Palaniswamy, 2024), in line with models of a shared prosodic-emotional network.

Neuroimaging studies reinforce the notion of overlapping emotional processing pathways for musical dissonance, emotional vocalisations, and affective images, with key limbic structures, such as the amygdala, consistently implicated across modalities (Koelsch, 2014; Frühholz et al., 2025). However, ERP topographies show differences specific to each modality, reflecting differential engagement of sensory-specific cortical regions (Olofsson et al., 2008; Filimonov et al., 2022; Proverbio et al., 2022). These findings align with Kölsch’s multilevel model of emotional processing of music, which posits that affective responses emerge through a hierarchy of neural processing phases, beginning with rapid subcortical evaluation and progressing gradually to more complex cortical evaluation (Koelsch, 2014). The initial ERP components observed in our study (e.g., N1 and P2) appear to represent automatic identification of affectively relevant features of the harmonic structure, consistent with subcortical processing. The later P3a, on the other hand, most likely represents higher-level cognitive appraisal and reorientation of attention to an affectively relevant stimulus. Thus, our ERP results support the hypothesis that emotional evaluation of musical consonances and dissonances is carried out through both hierarchically organised processes.

ERP components P2 and P3a, modulated by dissonant chords, resemble those observed in studies of emotional prosody and syntactic anomalies in speech (Friederici, 2011; Proverbio et al., 2020, 2022). This convergence lends support to the hypothesis of partially shared neural mechanisms for processing emotional salience in both music and language, a hypothesis that is consistent with models of a common prosodic-emotional network.

Our findings also agree with evidence of hemispheric asymmetries in music-induced emotion processing. Proverbio et al. (2020) demonstrated that consonant and dissonant chords activate different hemispheres—consonant sounds elicited stronger right temporal activation, whereas dissonant sounds preferentially activated left temporal regions. These asymmetries may reflect specialised processing of acoustic features (spectral vs. temporal resolution) and emotional valence in the auditory cortices.

We also found correlations between signal frequency characteristics and behavioural outcomes. The effects of the interaction between beta band amplitude (20–25 Hz) and dissonant stimuli indicate that high beta amplitude during stimulus presentation is associated with a significant reduction in the classification accuracy of dissonant chords. Specifically, a one-standard-deviation increase in beta amplitude corresponds to a 88% reduction in the odds of correctly identifying dissonant stimuli. This finding aligns with theories suggesting that beta oscillations are linked to the maintenance of current cognitive or perceptual sets and resistance to novel or deviant sensory input (Engel and Fries, 2010; Spitzer and Haegens, 2017). From this perspective, higher beta activity may indicate a perceptual condition in which the auditory system prioritises stability over flexibility, thereby reducing sensitivity to acoustically incongruent input, such as dissonance.

Two effects were observed for the gamma band amplitude (35–40 Hz). First, a main effect of stimulus type indicated that the probability of correctly identifying dissonant chords tended to be higher than that of consonant chords in the low gamma condition. However, this advantage appeared to be modulated by the amplitude of gamma: as gamma amplitude increased, the accuracy of detecting dissonant stimuli decreased markedly. In other words, dissonant chords were more discriminable at low gamma activity, but this advantage was weakened or reversed as gamma amplitude increased. Gamma oscillations have often been associated with attentional allocation, emotional salience, and perceptual binding (Başar-Eroglu et al., 1996; Mazaheri and Jensen, 2010). At the same time, excessive gamma activation may reflect heightened neural activity at the system level or network desynchronisation, particularly in response to emotional stimuli (Strube et al., 2021; Yu et al., 2022). Taken together, our results tentatively support the view that gamma activity contributes to stimulus evaluation but may reduce accuracy when over-amplified.

It should be noted that alpha band activity, often used in affective neuroscience as a metric for affective recovery and subjective preference, did not show a significant association with behavioural outcomes in our study. Although the alpha EEG is widely used in affective neuroscience due to its high reliability and reproducibility (Uusberg et al., 2013), its insensitivity in the present task might result from the sustained attention required during a relatively short (maximum running time of 16 min) randomised experimental session. Koelstra et al., for example, found a significant negative correlation between alpha EEG and valence, with higher arousal associated with lower alpha EEG amplitude when participants were exposed to music videos with varying emotional content. This interpretation is consistent with previous results indicating a decrease in alpha activity in task-focused conditions or during stimuli requiring evaluative processing (Koelstra et al., 2010; Paszkiel et al., 2020).

Several studies in the literature have shown a connection between neurodynamic EEG parameters and emotional responses (Choong et al., 2018). In our data, high-frequency LRTC (beta and gamma) showed associations primarily for trials categorised as “neutral.” However, the corresponding effect sizes were small in the beta range and unstable in the gamma range, suggesting that these findings should be interpreted with caution. One possible explanation is that neutral stimuli, unlike more emotionally charged ones, require more introspective processing, as reflected in both longer reaction times and lower accuracy. LRTC indices are believed to represent the balance between excitation and inhibition, as well as the temporal dynamics of information processing. These factors may be especially pertinent when participants try to classify a stimulus as neutral. Although preliminary, these findings suggest that LRTC may serve as a potential biomarker for distinguishing between neutral and affectively salient stimuli. However, this effect may also be related to the fact that the neutral type of stimulus was represented by the click of a metronome, the sound of which differed from the sound of the chords in timbre, as well as to the fact that the category “neutral” is less specific than “positive” or “negative.”

It is important to note several limitations of the study. First, the stimuli were synthesised using a single piano timbre. This ensured experimental control but limits generalisability, as timbre itself is a strong modulator of affective responses (Gabrielsson, 2001; Juslin and Västfjäll, 2008). Second, although the neutral condition served as a baseline, its acoustic structure (a single metronome click) was categorically different from consonant and dissonant chords. This choice ensured minimal affective salience, but it may also limit the direct comparability of the conditions and should be considered when interpreting the results. Third, the sample size and demographic homogeneity may restrict the generalisability of the findings. It should also be noted that all participants were non-musicians. Fourth, no preliminary registration was conducted to reduce the probability of Type I errors. Fifth, the odds ratio for some contrasts appeared numerically extreme with extensive CI. This is probably due to an imbalance in responses in some observations, rather than the actual size of the effect. Such inflated estimates are a known property of logistic models with rare events, and we therefore interpret them cautiously, focusing on the direction of the association rather than the absolute magnitude. Sixth, the use of categorical ratings (“pleasant,” “unpleasant,” “neutral”), rather than differentiated scales, limits the possibility of a more fine-grained interpretation of the effects. Whilst this approach ensured clear contrasts, it does not capture the full dimensional range of affective experience. Finally, the present findings were obtained in a Russian participant sample, and generalisation across cultures should be made with caution, given evidence that preferences for consonance and dissonance vary across populations (McDermott et al., 2016; Milne et al., 2023).

Importantly, although both consonant and dissonant chords were subjectively distinguished from neutral sounds, only dissonant chords were significantly distinguished at the level of EEG analysis. In the mid-latency (P2) and late (P3) intervals, dissonant chords showed widespread activation in frontal and temporoparietal regions, reflecting high affective salience associated with attention reorientation (Pallesen et al., 2005; Ellison et al., 2015). In contrast, consonant stimuli, although rated as pleasant at the behavioural level, failed to elicit comparably strong P2/P3 modulation under the same passive-listening conditions. This pattern is consistent with the negativity bias framework, according to which negatively valenced stimuli, across modalities, are prioritised in early perceptual and attentional processes because of their higher biological salience (Olofsson et al., 2008; Carretié, 2014), which partly explains the less striking neurophysiological response to positive valence in our data. At the same time, positive evaluations may involve later and more integrative processes, which are often modulated by cultural exposure and enculturation (McDermott et al., 2010; Argstatter, 2016). This temporal asymmetry may partly explain why consonant stimuli were behaviourally salient but less clearly differentiated in early ERP components. Whilst the unpleasant nature of dissonance is well-supported by multidisciplinary findings, the appealing characteristics of consonance apparently are the result of a complex interaction between cultural learning, memory, and specific cognitive and psychological mechanisms, in which culture plays a central role (Parncutt and Hair, 2018; Di Stefano and Spence, 2024). Thus, emotional valence in music is not merely a product of acoustic properties but deeply integrated with cognitive expectations and cultural backgrounds (Argstatter, 2016). For illustration, although infants universally demonstrate a preference for consonant intervals over dissonant ones (Trehub, 2003), certain isolated cultures exhibit no such preference for consonant chords (McDermott et al., 2010). This evidence suggests that culture has the capacity to modify innate preferences and elicit high-level emotional responses to harmonic structures. The “culture versus nature” debate has emphasised the interplay between psychoacoustic constraints (e.g., roughness and harmonicity) and enculturation (e.g., familiarity and context), as noted in complex music compositions, where dissonance can perform expressive or narrative functions without evoking unpleasant feelings (Juslin and Laukka, 2004). Key foundational studies advocate for composite models that integrate both sensory and cultural factors (Parncutt and Hair, 2018; see also Di Stefano and Spence, 2024, for recent re-examinations).

## 5 Conclusion

This experiment demonstrated how isolated piano timbre chords with varying harmonic structures elicit affective and neural responses. Behavioural outcomes demonstrated a reliable affective classification of consonant, dissonant, and neutral stimuli, confirming that musical consonance and dissonance correspond with subjective emotional valence.

Electrophysiological analysis revealed differential temporal properties and localisation of neural response sources between chord types. Dissonant and consonant chords elicited differential early activity in the temporal–parietal and auditory areas (90–110 ms), with the dissonant chords further displaying incised cortical activation at the middle latency (190–210 ms) and late (290–310 ms) time windows. Source localisation revealed the recruitment of parietal, frontal, and auditory areas, particularly in response to dissonant stimuli. Neural markers of perceptual sensitivity further complement these findings: elevated beta band amplitude (20–25 Hz) during the presentation of dissonant stimuli predicted significantly lower classification accuracy, suggesting reduced neural flexibility in processing incongruent input.

Additionally, increased gamma band amplitude (35–40 Hz) attenuated the behavioural advantage of dissonant chord recognition, highlighting a potential trade-off between salience-related gamma activation and perceptual precision. These findings suggest that musical harmony modulates both early perceptual and subsequent cognitive-affective stages in processing. Electrophysiological and behavioural responses to consonant and dissonant chords, as well as their comparison with neutral stimuli, support their potential usefulness as affective stimuli. However, further replication is required before chords can be considered established in this role. In addition to their potential utility for affective neuroscience, our findings underscore the promise of piano chords as stimuli for ERP research, particularly in cross-domain comparisons between music and language. Through their abstract form, chords can provide a controlled and versatile tool for investigating the general affective mechanisms underlying auditory communication.

## Data Availability

The datasets presented in this article are not readily available because the following investigations will be dependent on use of the data. Requests to access the datasets should be directed to Alexander Kirsanov, isbraintwister@gmail.com.
