# Supplementary material for: Electrophysiological and behavioural responses to consonant and dissonant piano chords as standardised affective stimuli
Source: Front Hum Neurosci. 2025 Oct 29;19:1689067. doi: 10.3389/fnhum.2025.1689067 (PMC12605063; doi:10.3389/fnhum.2025.1689067)
Supplement: Supplementary file 1 [file Data_Sheet_1.PDF]

Supplementary Table S1. Overview of empirical studies employing consonant and dissonant chords as stimuli.

Authors	Title	Idea	Disadvantages of the method
Malmberg, (1914)	The Perception of Consonance and Dissonance	Early empirical work linking pleasantness to simple frequency ratios and dissonance to harshness laid the foundation for later models of consonance.	Behavioural methodology only.
Plomp and Levelt, (1965)	Tonal Consonance and Critical Bandwidth	Quantitatively defines dissonance as intraband beating with maxima at certain frequency intervals, creating a reference psychoacoustic model. Simple frequency ratios sound more pleasant, complex ones less so; the concept of sensory dissonance (roughness) was introduced – maximum at intervals of ~1/6 tone.	Behavioural methodology only; large number of unstandartized stimuli (isolated tone pairs).

Regnault, Bigand & Besson (2001)	ERP evidence of consonance/dissonance processing	The central idea of the article is the empirical investigation of the distinct neural correlates underlying the processing advantage of consonant musical intervals over dissonant intervals, and the determination of how formal musical training modulates these differential brain responses.<br>The study addresses this by registering event-related brain potentials (ERPs)—specifically focusing on the Mismatch Negativity (MMN) component—to compare neural activations triggered by changes in a consonant context (consonance to dissonance) versus changes in a dissonant context (dissonance to consonance) among musicians and non-musicians. The ultimate goal is to describe the neural basis for the processing asymmetries along the consonance-dissonance continuum and demonstrate the influence of musical experience on the processing efficiency of consonant and dissonant stimul.	Neutral baseline validity. The interpretation of the addball paradigm data is questionable, where the presentation of the deviant is interpreted in terms of the features of the contextual stimulus. There is no assessment of the subjective perception of these stimuli.
Pallesen et al., (2005)	Emotion Processing of Major, Minor, and Dissonant Chords	This fMRI study examined automatic emotional responses to major, minor, and dissonant chords in musicians and nonmusicians. Passive listening to minor and dissonant chords elicited stronger BOLD responses in emotion-related regions, including the amygdala and brainstem, compared to major chords. These effects disappeared during a concurrent pitch working memory task, suggesting that cognitive demands attenuate affective reactivity. While musicians rated minor and dissonant chords as sadder and more unpleasant than nonmusicians, neural responses did not differ across groups, indicating that the initial affective response to chords is automatic and universal.	Chords were evaluated after the main series;<br>a slow neuroimaging technique was used;<br>neutral baseline validity.
Masataka, (2006)	Preference for consonance over dissonance by hearing newborns of deaf parents and of hearing parents	Infants aged 2–4 months prefer consonant sequences: they look at the source of the sound for longer and calm down. This preference is evident regardless of whether the parents are hearing or deaf, which indicates the intrinsic (innate) nature of consonance perception.	Specific participants sample;<br>behavioural methodology with indirect assessment.

Bidelman & Krishnan, (2009)	Neural Correlates of Consonance, Dissonance, and the Hierarchy of Musical Pitch in the Human Brainstem	This study examined brainstem correlates of consonance and dissonance, focusing on how basic pitch relationships are encoded in nonmusicians. Frequency-following responses revealed that consonant intervals elicited higher neural activity and pitch salience than dissonant intervals. Neural pitch salience corresponded both to the hierarchical tonal structure of Western music theory and to behavioural judgments of consonance.	Subcortical only; no conscious affect assessed.
Fritz et al., (2009)	Universal recognition of emotion in music	Cross-cultural study where compared Western listeners with the Mafa of Cameroon, who had no exposure to Western music. Mafa participants recognized happy, sad, and fearful emotions in Western music above chance, supporting the universality of basic emotion recognition. Both groups also preferred original over spectrally manipulated, dissonant versions, indicating that consonance and dissonance universally shape perceived pleasantness, though cultural experience modulates aesthetic judgments.	Behavioural methodology only; broad emotion categories only based on pictures.
Minati et al., (2009)	Functional MRI/Event-related potential study of sensory consonance and dissonance in musicians and nonmusicians	This study combined fMRI and ERP to examine neural correlates of sensory consonance and dissonance in musicians and nonmusicians. Consonant chords evoked stronger fMRI responses in prefrontal regions, with greater right-lateralization in nonmusicians and more symmetric activation in musicians. ERP results showed P1 modulation by consonance in both groups, indicating a shared early processing stage, while the N2 component was enhanced only in musicians, suggesting an analytic strategy contrasting with the nonmusicians' affective response. These findings point to differential reliance on emotional versus cognitive mechanisms in music processing across expertise levels.	Emotional assessments are not used.

McDermott et al., (2010)	Individual differences reveal the basis of consonance	This study addressed the origins of consonance and dissonance by testing acoustic accounts based on beating versus harmonicity. Individual differences showed that preference for harmonic spectra, but not reduced beating, predicted a stronger liking for consonant chords. Preference for harmonicity also correlated positively with musical experience, suggesting that exposure enhances aesthetic sensitivity to natural harmonic frequency relations, which constitute the perceptual foundation of consonance.	A rough assessment of the participant's musical experience; only behavioural metodology: cultural exposure vs. innate bias unresolved.
Dellacherie et al.,(2011)	The effect of musical experience on emotional self-reports and psychophysiological responses to dissonance	This study examined how musical experience shapes emotional responses to dissonance, combining self-reports with measures of skin conductance, heart rate, and facial electromyography. Dissonance was rated as more unpleasant and produced stronger physiological reactions in highly experienced listeners. Within a defense cascade framework, results indicated that training amplifies aversion to dissonance, affecting both autonomic activity and expressive somatic reactions, including increased zygomatic muscle activity associated with negative valence. Findings highlight the role of learned aesthetic preference in modulating internal and expressive emotional responses to music.	Limited stimulus material (lack of standartisaion); limited neurophysiological depth.
Virtala et al., (2014)	Musicianship facilitates the processing of Western music chords—An ERP and behavioral study	This EEG study examined how musicianship influences neural and behavioral processing of Western chords (major, minor, inverted major). Musicians showed larger MMN and N1 amplitudes and outperformed nonmusicians in chord discrimination, indicating superior pre-attentive and attentive processing. While nonmusicians exhibited subtle neural differences for basic categories (major vs. minor), only musicians reliably detected complex distinctions such as inversions. These results suggest that enhanced chord processing reflects specialized neural adaptations from explicit musical training rather than general cognitive advantages.	Task/attention confounds: only consonant chords; oddball paradigm without emotional ranking; between group design.

Foo et al., (2016)	ECoG of superior temporal gyrus	This 2016 ECoG study (Foo et al.) examined neural processing of consonance and dissonance in the superior temporal gyrus (STG). High-resolution recordings from neurosurgical patients revealed increased high-gamma activity for dissonant chords shortly after onset, with a distinct anterior–posterior organization in the right STG. The findings suggest that cortical sensitivity to dissonance reflects the acoustic roughness of these chords, highlighting early and spatially specific encoding of affectively salient harmonic structures.	Invasive; clinical patients only.
Bianco et al., (2016)	Networks for harmonic structure	This fMRI study with expert pianists examined neural networks for harmonic structure in music perception and action. Results revealed a dissociation between a dorsal fronto-parietal network engaged during action imitation and a ventral fronto-temporal network active during passive listening. Both pathways recruited distinct subregions of the right inferior frontal gyrus, suggesting its role as a hub for integrating harmonic information across modalities. Findings support a predictive coding account, with long-term harmonic knowledge represented in modality-specific posterior areas that interact with frontal regions to optimize predictions in both perception and motor planning.	Sequences, not isolated chords.
Pagès-Portabella et al., (2020)	Dissonant endings of chord progressions elicit a larger ERAN than ambiguous endings in musicians	This study examined neural responses to harmonic expectation violations in musicians and nonmusicians. Using ERP, the researchers presented chord sequences ending with either mild (consonant but ambiguous) or strong (dissonant) violations. Both groups showed an early right anterior negativity (ERAN) to irregular endings, indicating automatic detection of syntactic violations. However, only musicians exhibited larger ERAN amplitudes for dissonant versus mild violations, suggesting that training enhances neural sensitivity to the degree of harmonic irregularity.	Musical expertise / culture; Task/attention confounds. Focus on harmonic syntax may conflate dissonance with expectancy violation.

Lahdelma & Eerola, (2020)	Cultural and musical training effects on chord consonance judgments	This study examined how cultural familiarity and musical expertise shape perceptions of consonance and dissonance. In Experiment 1, strong correlations were found among perceptual concepts such as pleasantness, preference, and tension, with effects amplified in musicians but influenced by timbre and pitch number. Experiment 2 showed that cultural familiarity significantly modulated these relationships for both musicians and nonmusicians. Across conditions, tension emerged as the most reliable proxy for consonance–dissonance perception. The authors argue for distinguishing related concepts and emphasize the need to control for cultural background and expertise in future research.	Online paradigm reduces experimental control; self-reported expertise.
