# Supplementary material for: Electrophysiological and behavioural responses to consonant and dissonant piano chords as standardised affective stimuli
Source: Front Hum Neurosci. 2025 Oct 29;19:1689067. doi: 10.3389/fnhum.2025.1689067 (PMC12605063; doi:10.3389/fnhum.2025.1689067)
Supplement: Supplementary file 2 [file Data_Sheet_2.PDF]

Supplementary Table S2. Post-hoc pairwise comparisons of reaction times across stimulus categories.

group1	group2	n1	n2	statistic	p	p.adj	p.adj.signif
dissonant incongruent	consonant incongruent	522	464	-2.0	0.045	0.090	ns
dissonant incongruent	neutral incongruent	522	747	-7.11	1.12 × 10^-12	1.01 × 10^-11	****
dissonant incongruent	dissonant congruent	522	1123	-10.43	1.79 × 10^-25	2.33 × 10^-24	****
dissonant incongruent	consonant congruent	522	1158	-9.64	5.39 × 10^-22	6.47 × 10^-21	****
dissonant incongruent	neutral congruent	522	688	-14.02	1.21 × 10^-44	1.81 × 10^-43	****
consonant incongruent	neutral incongruent	464	747	-4.7	2.56 × 10^-6	1.28 × 10^-5	****
consonant incongruent	dissonant congruent	464	1123	-7.7	1.41 × 10^-14	1.41 × 10^-13	****
consonant incongruent	consonant congruent	464	1158	-6.92	4.43 × 10^-12	3.54 × 10^-11	****
consonant incongruent	neutral congruent	464	688	-11.42	3.47 × 10^-30	4.85 × 10^-29	****
neutral incongruent	dissonant congruent	747	1123	-3.11	0.002	0.008	**
neutral incongruent	consonant congruent	747	1158	-2.18	0.029	0.087	ns
neutral incongruent	neutral congruent	747	688	-7.72	1.19 × 10^-14	1.30 × 10^-13	****
dissonant congruent	consonant congruent	1123	1158	01.06	0.290	0.290	ns
dissonant congruent	neutral congruent	1123	688	-5.39	6.92 × 10^-8	4.15 × 10^-7	****
consonant congruent	neutral congruent	1158	688	-6.35	2.22 × 10^-10	1.55 × 10^-9	****

Values indicate group sample sizes (n1, n2), Wilcoxon rank-sum test statistics, uncorrected p-values (p), Holm-adjusted p-values (p.adj), and corresponding significance codes (*ns, *, **, ***, *****). P-values are reported to three decimal places or in scientific notation (e.g., 1.12 × 10^–12) where appropriate.
