# Supplementary material for: Electrophysiological and behavioural responses to consonant and dissonant piano chords as standardised affective stimuli
Source: Front Hum Neurosci. 2025 Oct 29;19:1689067. doi: 10.3389/fnhum.2025.1689067 (PMC12605063; doi:10.3389/fnhum.2025.1689067)
Supplement: Supplementary file 3 [file Data_Sheet_3.PDF]

Supplementary Table S3. Post-hoc pairwise comparisons of classification accuracy across consonant, dissonant, and neutral chords.

contrast	group1	group2	category	n_g1_focus	n_g1_other	n_g2_focus	n_g2_other	OR	CI_low	CI_high	p_raw	p_adj_holm
consonant vs dissonant	consonant	dissonant	pleasant	1158	464	356	1289	09.03	7.68	10.63	< .0001	< .0001
consonant vs neutral	consonant	neutral	pleasant	1158	464	444	991	5.57	4.75	6.53	< .0001	< .0001
dissonant vs neutral	dissonant	neutral	unpleasant	1123	522	303	1132	08.03	6.8	9.5	< .0001	< .0001
dissonant vs consonant	dissonant	consonant	unpleasant	1123	522	252	1370	11.69	9.84	13.92	< .0001	< .0001
neutral vs consonant	neutral	consonant	neutral	688	747	212	1410	6.12	5.11	7.35	< .0001	< .0001
neutral vs dissonant	neutral	dissonant	neutral	688	747	166	1479	8.2	6.76	9.99	< .0001	< .0001

Reported are contrasts between stimulus categories and response categories (pleasant, unpleasant, neutral). The table includes sample sizes for each group (n_g1_focus, n_g1_other, n_g2_focus, n_g2_other), odds ratios (OR) with 95% confidence intervals (CI_low, CI_high), uncorrected p-values (p_raw), Holm-adjusted p-values (p_adj_holm), and corresponding significance levels. p-values are reported to four decimal places or as p < .0001 when smaller.
