# Supplementary material for: Electrophysiological and behavioural responses to consonant and dissonant piano chords as standardised affective stimuli
Source: Front Hum Neurosci. 2025 Oct 29;19:1689067. doi: 10.3389/fnhum.2025.1689067 (PMC12605063; doi:10.3389/fnhum.2025.1689067)
Supplement: Supplementary file 4 [file Data_Sheet_4.PDF]

Supplementary Table S4. Results and model characteristics from mixed-effects logistic regression analyses: beta band (20–25 Hz).

Predictor	β (Estimate)	SE	z	p	OR	95% CI (OR)	β* (std.)
(Intercept)	0.75	0.65	1.15	.250	2.12	[0.59, 7.6]	–
Stimulus: Neutral	-0.94	1.21	-0.78	.437	0.39	[0.037, 4.2]	–
Stimulus: Dissonant	1.96	01.01	1.94	.053·	07.09	[0.98, 51]	–
Beta amplitude (20–25 Hz)	0.32	0.61	0.52	.603	1.37	[0.42, 4.5]	0.11
Neutral × Beta amplitude	-0.58	1.12	-0.52	.604	0.56	[0.063, 5]	–
Dissonant × Beta amplitude	-2.08	0.94	-2.22	.027*	0.12	[0.02, 0.78]	-0.73

Notes.
OR = odds ratio, CI = Wald 95% confidence interval.
β* = standardized coefficient.

Model fit: AIC = 5378.9, BIC = 5457.2, logLik = –2677.4.
Marginal R^2 = 0.11, Conditional R^2 = 0.43, Tjur's R^2 = 0.29, AUC = 0.82.

Random effects: variance of intercepts (participants) = 1.26; variance of slopes (stimulus type) = 4.54; ICC = 0.36.

Diagnostics: no overdispersion (DHARMa p = .91), no uniformity violation (p = .26), VIFs up to 103.9.

LR Tests with AIC and BIC

it_null: AIC = 5390.8, BIC = 5436.5, logLik = –2688.4
it_main: AIC = 5379.7, BIC = 5445.0, logLik = –2679.9
it_full: AIC = 5378.9, BIC = 5457.2, logLik = –2677.4
LR: it_null vs it_main → $\chi^2(3)$ = 17.06, $p < .001$
LR: it_main vs it_full → $\chi^2(2)$ = 4.85, p = .088
