# Supplementary material for: Electrophysiological and behavioural responses to consonant and dissonant piano chords as standardised affective stimuli
Source: Front Hum Neurosci. 2025 Oct 29;19:1689067. doi: 10.3389/fnhum.2025.1689067 (PMC12605063; doi:10.3389/fnhum.2025.1689067)
Supplement: Supplementary file 5 [file Data_Sheet_5.PDF]

Supplementary Table S5. Results and model characteristics from mixed-effects logistic regression analyses: gamma band (35–40 Hz).

Predictor	β (Estimate)	SE	z	p	OR	95% CI (OR)	β* (std.)
(Intercept)	0.54	0.57	0.96	.339	1.72	[0.57, 5.2]	–
Stimulus: Neutral	-0.87	01.05	-0.83	.408	0.42	[0.053, 3.3]	–
Stimulus: Dissonant	2.13	0.84	2.52	.012*	8.41	[1.6, 44]	–
Gamma amplitude (35–40 Hz)	01.01	1	01.01	.311	2.76	[0.39, 20]	0.21
Neutral × Gamma amplitude	-1.25	1.84	-0.68	.497	0.29	[0.0077, 11]	–
Dissonant × Gamma amplitude	-4.37	1.49	-2.93	.003**	0.01	[0.00068, 0.23]	-0.92

Notes.
OR = odds ratio, CI = Wald 95% confidence interval.
β* = standardized coefficient.

Model fit: AIC = 5376.0, BIC = 5454.3, logLik = –2676.0.
Marginal R^2 = 0.11, Conditional R^2 = 0.42, Tjur's R^2 = 0.29, AUC = 0.82.

Random effects: variance of intercepts (participants) = 1.23; variance of slopes (stimulus type) = 4.52; ICC = 0.35.

Diagnostics: no overdispersion (DHARMa p = .86), no uniformity violation (p = .50), VIFs up to 64.4.

LR Tests with AIC and BIC

it_null: AIC = 5390.8, BIC = 5436.5, logLik = –2688.4
it_main: AIC = 5382.5, BIC = 5447.7, logLik = –2681.2
it_full: AIC = 5372.7, BIC = 5451.0, logLik = –2674.3
LR: it_null vs it_main → $\chi^2(3) = 14.32$, p = .0025
LR: it_main vs it_full → $\chi^2(2) = 13.79$, p = .0010
