# Supplementary material for: Electrophysiological and behavioural responses to consonant and dissonant piano chords as standardised affective stimuli
Source: Front Hum Neurosci. 2025 Oct 29;19:1689067. doi: 10.3389/fnhum.2025.1689067 (PMC12605063; doi:10.3389/fnhum.2025.1689067)
Supplement: Supplementary file 6 [file Data_Sheet_6.PDF]

Supplementary Table S6. Results and model characteristics from mixed-effects logistic regression analyses: beta DFA (20–25 Hz).

**Predictor**	**β (Estimate)**	**SE**	**z**	**p**	**OR**	**95% CI (OR)**	**β* (std.)**
(Intercept)	2.71	1.51	1.79	.073·	15.02	[0.78, 290]	–
Stimulus: Neutral	3.5	2.57	1.36	.174	33.10	[0.21, 5100]	–
Stimulus: Dissonant	-2.65	2.48	-1.07	.285	0.07	[0.00055, 9.1]	–
Beta DFA (20–25 Hz)	-2.65	2.43	-1.09	.275	0.07	[0.00061, 8.2]	-0.19
Neutral × Beta DFA	-8.1	4.13	-1.96	.050·	0.00	[9.1e-08, 1]	–
Dissonant × Beta DFA	04.04	3.98	01.02	.310	56.68	[0.023, 140000]	0.29

Notes.
OR = odds ratio, CI = Wald 95% confidence interval.
β* = standardized coefficient.

Model fit: AIC = 5379.2, BIC = 5457.5, logLik = –2677.6.
Marginal R^2 = 0.12, Conditional R^2 = 0.42, Tjur's R^2 = 0.29, AUC = 0.82.

Random effects: variance of intercepts (participants) = 1.24; variance of slopes (stimulus type) = 4.14; ICC = 0.34.

Diagnostics: no overdispersion (DHARMa p = .78), no uniformity violation (p = .58), VIFs up to 2641.7.

LR Tests with AIC and BIC

it_null: AIC = 5390.8, BIC = 5436.5, logLik = –2688.4
it_main: AIC = 5381.3, BIC = 5446.5, logLik = –2680.7
it_full: AIC = 5379.2, BIC = 5457.5, logLik = –2677.6
LR: it_null vs it_main → $\chi^2(3)$ = 15.49, p = .0014
LR: it_main vs it_full → $\chi^2(2)$ = 6.06, p = .048
