# Supplementary material for: Electrophysiological and behavioural responses to consonant and dissonant piano chords as standardised affective stimuli
Source: Front Hum Neurosci. 2025 Oct 29;19:1689067. doi: 10.3389/fnhum.2025.1689067 (PMC12605063; doi:10.3389/fnhum.2025.1689067)
Supplement: Supplementary file 7 [file Data_Sheet_7.PDF]

Supplementary Table S7. Results and model characteristics from mixed-effects logistic regression analyses: gamma DFA (35–40 Hz).

**Predictor**	**β (Estimate)**	**SE**	**z**	**p**	**OR**	**95% CI (OR)**	**β* (std.)**
(Intercept)	1.58	1.25	1.26	.206	4.86	[0.42, 56]	–
Stimulus: Neutral	4.88	02.03	2.4	.016*	131.63	[2.4, 7100]	–
Stimulus: Dissonant	-2.14	02.03	-1.05	.293	0.12	[0.0022, 6.4]	–
Gamma DFA (35–40 Hz)	-0.77	1.88	-0.41	.682	0.46	[0.012, 19]	-0.08
Neutral × Gamma DFA	-9.74	03.07	-3.17	.002**	0.00	[1.4e-07, 0.024]	–
Dissonant × Gamma DFA	03.02	03.06	0.99	.324	20.50	[0.051, 8300]	0.30

Notes.
OR = odds ratio, CI = Wald 95% confidence interval.
β* = standardized coefficient.

Model fit: AIC = 5372.7, BIC = 5451.0, logLik = –2674.3.
Marginal R^2 = 0.15, Conditional R^2 = 0.42, Tjur's R^2 = 0.29, AUC = 0.82.

Random effects: variance of intercepts (participants) = 1.27; variance of slopes (stimulus type) = 3.64; ICC = 0.33.

Diagnostics: no overdispersion (DHARMa p = .96), no uniformity violation (p = .55), VIFs up to 1361.3.

LR Tests with AIC and BIC

it_null: AIC = 5390.8, BIC = 5436.5, logLik = –2688.4
it_main: AIC = 5382.5, BIC = 5447.7, logLik = –2681.2
it_full: AIC = 5372.7, BIC = 5451.0, logLik = –2674.3
LR: it_null vs it_main → $\chi^2(3)$ = 14.32, p = .0025
LR: it_main vs it_full → $\chi^2(2)$ = 13.79, p = .0010
